# The statistics of epidemic transitions

**DOI:** 10.1371/journal.pcbi.1006917

**Published:** 2019-05-08

**Authors:** John M. Drake, Tobias S. Brett, Shiyang Chen, Bogdan I. Epureanu, Matthew J. Ferrari, Éric Marty, Paige B. Miller, Eamon B. O’Dea, Suzanne M. O’Regan, Andrew W. Park, Pejman Rohani

**Affiliations:** 1 Odum School of Ecology, University of Georgia, Athens, Georgia, United States of America; 2 Center for the Ecology of Infectious Diseases, University of Georgia, Athens, Georgia, United States of America; 3 Department of Mechanical Engineering, University of Michigan, Ann Arbor, Michigan, United States of America; 4 Automotive Research Center, University of Michigan, Ann Arbor, Michigan, United States of America; 5 Center for Infectious Disease Dynamics, Pennsylvania State University, State College, Pennsylvania, United States of America; 6 Department of Mathematics, North Carolina A&T State University, Greensboro, North Carolina, United States of America; ETH Zurich, SWITZERLAND

## Abstract

Emerging and re-emerging pathogens exhibit very complex dynamics, are hard to model and difficult to predict. Their dynamics might appear intractable. However, new statistical approaches—rooted in dynamical systems and the theory of stochastic processes—have yielded insight into the dynamics of emerging and re-emerging pathogens. We argue that these approaches may lead to new methods for predicting epidemics. This perspective views pathogen emergence and re-emergence as a “critical transition,” and uses the concept of noisy dynamic bifurcation to understand the relationship between the system observables and the distance to this transition. Because the system dynamics exhibit characteristic fluctuations in response to perturbations for a system in the vicinity of a critical point, we propose this information may be harnessed to develop early warning signals. Specifically, the motion of perturbations slows as the system approaches the transition.

## Anticipating epidemic transitions

Outbreaks of re-emerging pathogens are among the most unpredictable threats to public health and global security [1]. In recent years, epidemics of measles, mumps, polio, whooping cough and other vaccine-preventable diseases have caused death and disease [2, 3], captured headlines and focused political attention, and prompted substantial investment in emergency planning and preparedness in both developed and developing countries. The causes of pathogen re-emergence (as well as the emergence of new pathogens) are variable and seemingly idiosyncratic [4]. For this reason, predicting their eruption might seem intractable [5]. Here we question this pessimism and suggest instead that data-driven methods based on the characteristic fluctuations of near-critical systems may provide model-independent measurements of the approach to disease criticality before a crisis occurs.

The approach we propose anticipates disease re-emergence through a *critical transition*, *i.e*. when driving factors such as pathogen evolution, spatial movement, or (most central to the argument in this paper) changes in vaccine uptake cause the system to drift toward the critical point where transmission is sustained. Following Scheffer [6], we define a critical transition as a phenomenon in which a small perturbation to a system’s state or a small change in one of its parameters triggers a cycle of positive feedback that results in a regime shift, *i.e*., a qualitative change in the dynamics of the system, holistically characterized in terms of attracting points, stochastic fluctuations, and cycles. Critical transitions are to be contrasted with regime shifts that are a direct consequence of large, external shocks. Critical transitions have also been considered in terms of bifurcations of dynamical systems, which provides a useful way to think about the behaviors of near-critical systems, like re-emerging infectious diseases [7]. Most importantly, neither the location of the critical transition nor the state of the system is perfectly known. It is thus the consistent behavior of diverse models near bifurcation that provides the model-independent basis for predicting critical transitions.

In contrast to most research on critical transitions, which concern the catastrophic shifts associated with saddle-node bifurcations, epidemic transitions are associated with a transcritical bifurcation, which is not abrupt, but piecewise continuous. That is, when the mean prevalence moves above zero in response to a small change in parameters, it nonetheless remains close to zero. Therefore, there can be no rapid shift to a distant equilibrium. However, even these incremental increases in theoretical prevalence are important due to stochastic effects when the pathogen is introduced at a low rate from an external source. This is because even if each infectious case can, on average, generate more than one additional case, there is a non-zero probability of the chain of infections going extinct before an epidemic occurs [8]. Therefore, the state of the stochastic system may remain close to zero prevalence even as the stable equilibrium value of prevalence predicted by the deterministic model increases. By the time an epidemic occurs, the stable equilibrium may be distant from zero and it becomes appropriate to consider the rapid change in the state of the system over the course of the outbreak as a critical transition. Particularly, Dibble *et al*. [8] showed that for a simple model of disease emergence, epidemics moving to a distant stable equilibrium were typical of a wide range of parameter values.

Our goal is to find statistical methods to anticipate epidemics. The point in connecting outbreak potential with bifurcations is that the theory of near-critical systems predicts that dynamics will exhibit consistent features, stemming from the phenomenon of *critical slowing down* [6, 7, 9]. This is distinct from effects of individual heterogeneity or movement on the existence and location of the critical threshold [10, 11]. As is well known, in the vicinity of a bifurcation, critical slowing down can be understood mathematically through stability analysis [12]. In continuous-time and for frequent perturbations, the transmission process may be approximately represented by a system of stochastic differential equations, which can be studied using a linear Fokker-Planck equation, the solution of which is a Gaussian distribution [13]. Such analysis shows correlations for this process to decay exponentially, with characteristic timescales given by the reciprocals of the eigenvalues of the drift matrix [13]. At the transition, the drift matrix in the Fokker-Planck equation becomes singular, implying that perturbations in (or near) one direction will persist indefinitely. Various statistically measurable properties may arise from the increasing persistence of perturbations. For instance, from the Fokker-Planck equation it can also be shown that the second moment of fluctuations in the dominant eigendirection diverges as an eigenvalue approaches zero. In data generated by such a process, this would be manifested as an increase in sample variance. Such an empirical early warning signal therefore provides evidence that the system is approaching a critical transition.

## Critical slowing down in a contagion process with vaccination

**Fig 1:**
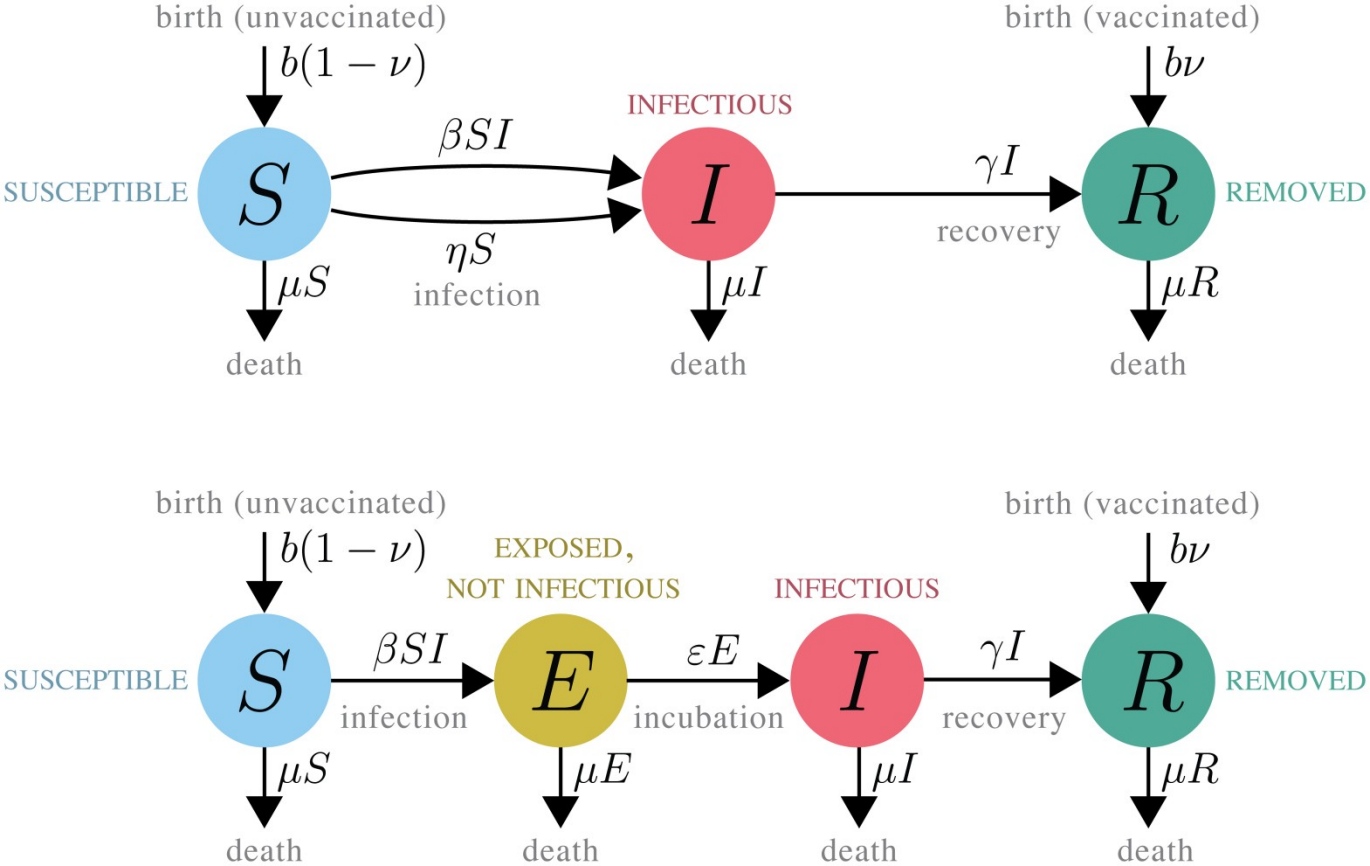
The SIR and SEIR models.

Critical slowing down is illuminated by a closer look at a theoretical contagion process. We start with the standard model of mathematical epidemiology, the general epidemic or SIR model [14, 15], shown schematically in Fig 1. A finite population with a fixed expected size is divided into classes of Susceptible, Infected, and Removed individuals, denoted *S*, *I*, and *R*, respectively. Variables affecting flow from one class to another are the transmission rate *β*, the “sparking rate” *η* (which mimics the transmission from outside sources), the recovery rate *γ*, the birth rate *b*, the fraction of births that are immunized by vaccination *ν*, and the death rate *μ* (Fig 1). Because the population size is finite, the dynamics are subject to demographic stochasticity [16]. An approximate description of the dynamics of the susceptible and infected classes is given by the stochastic differential equations

$$\begin{array}{l} \frac{\text{d}S}{\text{d}t}=-\beta SI-\eta S+b\left(1-\nu \right)-\mu S+w_{S}\left(t\right),\\ \frac{\text{d}I}{\text{d}t}=\beta SI+\eta S-\left(\gamma +\mu \right)I+w_{I}\left(t\right), \end{array}$$ (1)

where *w*_*S*_(*t*) and *w*_*I*_(*t*) are zero mean Gaussian white noise sources with covariance matrix

$$\begin{array}{c} B=[\begin{array}{cc} \beta SI+\eta S+b(1-\nu )+\mu S & -(\beta SI+\eta S)\\ -(\beta SI+\eta S) & \beta SI+\eta S+(\gamma +\mu )I \end{array}]. \end{array}$$ (2)

Despite their simplicity, such models have provided profound insight into many features of infectious disease dynamics and contagion processes in general [14]. An extension of this model, accommodating an infected-but-not-infectious state, is the SEIR model (Fig 1).

Interestingly, the SEIR model predicts with remarkable accuracy the period of oscillations apparent in time series of disease incidence for a number of well-known infectious diseases [17]. Table 1 reproduces the classic finding of Anderson and May [14] showing agreement between predicted and observed periods of oscillation for measles, rubella, mumps, poliomyelitis, smallpox, chickenpox, scarlet fever, diphtheria, and pertussis from a variety of eras and locations (see also Ref. 17). For these disease systems, in which the host lifespan greatly exceeds the infectious period (*μ* ≪ *γ*), the number infected in the model exhibits oscillations with a natural period of approximately $2\pi \sqrt{AD}$, where *A* is the average age at which the infection is acquired and *D* is the expected duration of the infection (*i.e*., the sum of the latent and infectious periods) [18]. The predictions are thus based on empirical estimates of *A* and *D* and the agreement between theory and data provides evidence that these simple models capture the main rules that govern many infectious disease systems. As we shall show, this success also provides empirical evidence for the premise that the speed of the dynamics of an infectious disease system indicates proximity to the immunization threshold.

**Table 1:** Inter-epidemic periods of some common infections.

Infection	Setting	Calculated	Observed
Measles	England and Wales, 1948–68	2	2
	Aberdeen, Scotland, 1883–1902	2	2
	Baltimore, USA, 1900–27	2	2
	Paris, France, 1880–1910	2	2
	Yaounde, Cameroun, 1968–75	1–2	1
Rubella	Manchester, UK, 1916–83	4–5	3.5
	Glasgow, Scotland, 1929–64	4–5	3.5
Mumps	England and Wales, 1948–82	3	3
	Baltimore, USA, 1928–73	3–4	2–4
Poliomyelitis	England and Wales, 1948–65	4–5	3–5
Smallpox	India, 1868–1948	4–5	5
Chickenpox	New York City, USA, 1928–72	3–4	2–4
	Glasgow, Sotland, 1929–72	3–4	2–4
Scarlet fever	England and Wales, 1897–1978	4–5	3–6
Diphtheria	England and Wales, 1897–1979	4–5	4–6
Pertussis	England and Wales, 1948–85	3–4	3–4

Theoretical and observed inter-epidemic periods (in years) of some common infections (from Table 6.1 of Ref. 14).

**Fig 2:**
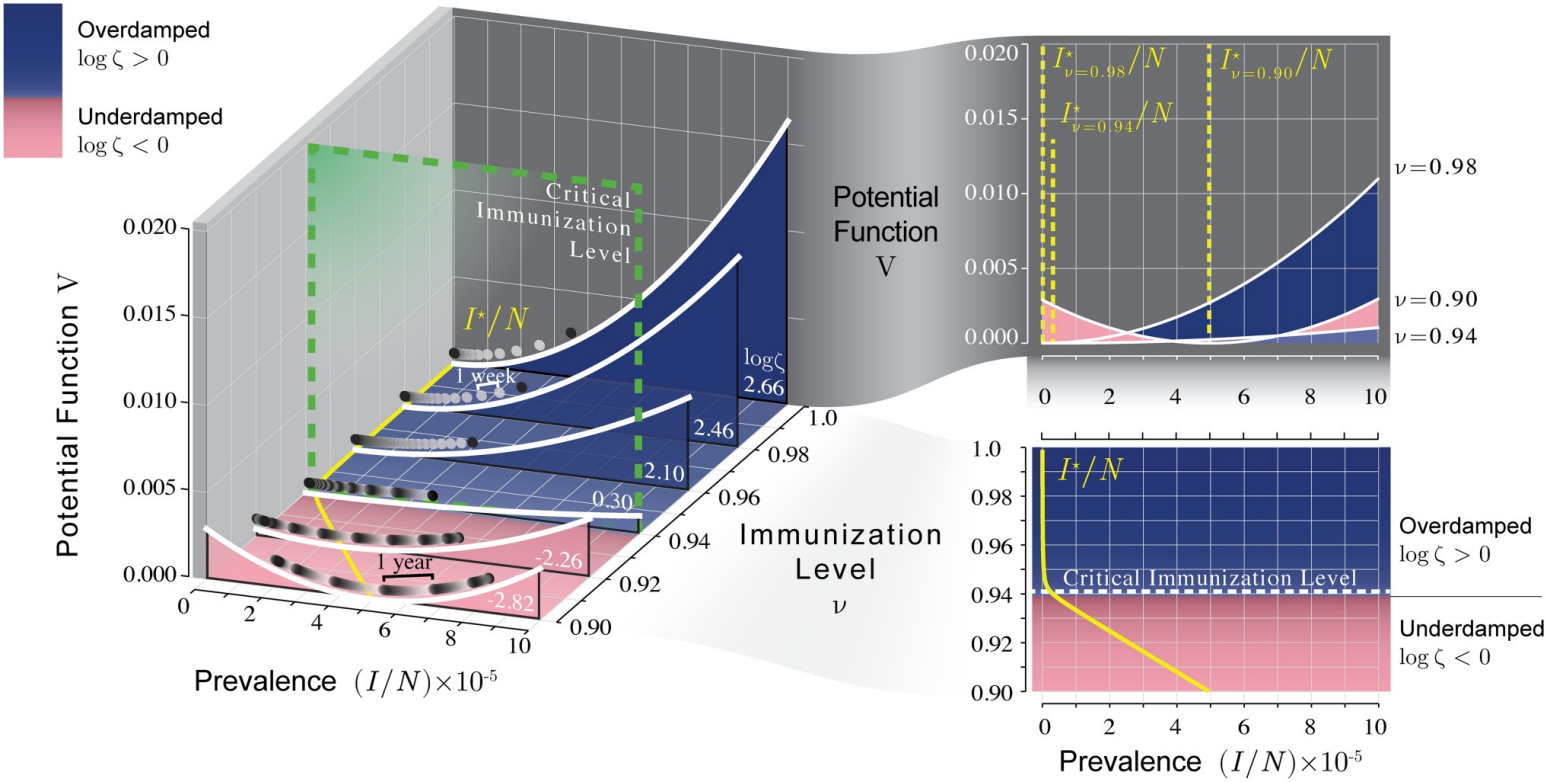
Critical slowing down is illustrated in the potential function of the linearized SIR model. Disease prevalence (*I*/*N*) is represented by the horizontal position of a ball sliding through viscous fluid in a well having a height determined by the potential function. Both the depth of the well and the viscosity of the fluid in the equivalent physical system are affected by vaccine coverage. The well is shallowest near the immunization threshold, which illustrates the slowing down of the dynamics as the critical point (*ν*_*c*_ ≈ 0.941; green dashed line) is approached. Oscillatory dynamics occur at another immunization level (*ν* ≈ 0.939) corresponding to the system becoming underdamped (pink region). Model parameters: *b* = 2 × 10^5^ y^−1^, *μ* = 0.02 y^−1^, *γ* = 365/22 y^−1^, *η* = 2 × 10^−5^ y^−1^, *R*_0_ = 17. To write the potential function in terms of prevalence, we scaled the deviations of the linearized system by the square root of the equilibrium population size (*i.e*., scaled by $\sqrt{b/\mu }$). Critical slowing down is seen in this figure in the relative magnitude of the displacement of the ball with respect to the distance from the critical level of immunization.

For our theory, the essential link between the dynamics of the disease and the proximity to control by vaccination lies in the stability of its equilibrium. For trajectories that begin in the neighborhood of the equilibrium, we simplify the equations by linearizing them [12]. The solutions of the linear system are equivalent to those of a damped harmonic oscillator [15], and thus one can draw on well-known equivalent physical systems, such as a mass on a spring, to understand the dynamics. Fig 2 illustrates this phenomenon by analogy to a ball sliding in a well for various levels of the vaccine uptake. The increased depth of the well corresponds to increased stability of the equilibrium. In Fig 2 the wells become more shallow as the vaccine uptake moves closer to the threshold. Balls placed a given distance to the right of the bottom of the wells in the diagram will move most slowly in the well that is closest to the immunization threshold.

## Stability of the endemic equilibrium

Insight into the dynamics of the SIR model (Eq 1) is obtained through linear stability analysis. As is well known, in the vicinity of a bifurcation, critical slowing down can be understood mathematically through linear stability analysis [12]. When a system is perturbed away from a stable equilibrium, the perturbations decay with a rate determined by the eigenvalues of the linearized system. The condition for an equilibrium to be stable in a continuous-time system is that all eigenvalues have negative real component (or are less than unity for a discrete-time system); a bifurcation occurs when this condition is violated [12]. As the model parameters vary, the approach to the bifurcation is characterized by an increase in one or more of the eigenvalues. In the neighborhood of the transition, the dominant eigenvalue (the eigenvalue with largest real part) becomes increasingly close to zero (one for discrete-time dynamics) [12]. The fluctuations about the equilibrium, ***z***(*t*) = (*z*_*S*_, *z*_*I*_), solve the system of linear equations,

$$\begin{array}{c} \frac{\text{d}z}{\text{d}t}=\text{J}z, \end{array}$$ (3)

where the matrix **J** is the Jacobian evaluated at the equilibrium. By differentiating the second equation in Eq 3 with respect to time and eliminating *z*_*S*_, one obtains

$$\frac{{\text{d}}^{2}z_{I}}{\text{d}t^{2}}-\tau \frac{\text{d}z_{I}}{\text{d}t}+\Delta z_{I}=0,$$ (4)

where *τ* and Δ respectively denote the trace and determinant of the Jacobian matrix. The second order linear differential equation, Eq 4, describes the dynamics of a damped harmonic oscillator [19]. Following this physical analogy, the potential at point *z*_*I*_ is found by integrating the restorative force, $V\left(z_{I}\right)=\frac{\Delta z_{I}^{2}}{2}$. Changes in Δ result in changes to the depth of the wells in Fig 2. Another informative quantity is the damping ratio $\zeta =-\tau /\sqrt{4\Delta }$. A damping ratio of less than one indicates that the trajectory of *z*_*I*_ will oscillate as it approaches zero. The equation for *z*_*I*_ is *z*_*I*_ (*t*) = *k*_1_ exp(λ_1_*t*) + *k*_2_ exp(λ_2_*t*) where λ_1_ and λ_2_ are the eigenvalues of **J** and *k*_1_ and *k*_2_ are determined by the initial values of *z*_*I*_ and *z*_*S*_.

Equations for the eigenvalues of the Jacobian provide a precise statement of the relationship between the distance of the vaccine uptake to the immunization threshold and the speed of the dynamics. This fact might be useful in practical applications where the distance to the threshold is unknown. For the SIR model, it is instructive to consider the equations in the limiting case that the sparking rate *η* = 0, recognizing that for small *η* the eigenvalues will differ only slightly [20]. Let *N* = *b*/*μ* be the expected total population size. When *η* = 0, for high enough *ν*, the Jacobian at the disease-free equilibrium of (S*, I*) = (*N*(1 − *ν*), 0) has the eigenvalues of −*μ* and (*γ* + *μ*)[*R*_0_(1 − *ν*) − 1], where the basic reproduction number, *R*_0_ = *Nβ*/(*γ* + *μ*), is a controlling parameter for the model in the absence of vaccination (*i.e*., when *ν* = 0). In the presence of vaccination, the controlling parameter is the product *R*_0_(1 − *ν*). To see why, note that when *R*_0_(1 − *ν*) < 1, both of the eigenvalues of the disease-free equilibrium are negative and thus small deviations from the disease-free equilibrium decay exponentially over time. Hence, introduction of the disease will not lead to an epidemic or the disease persisting endemically. Rather, transmission is prevented by vaccination. In contrast, when *R*_0_(1 − *ν*) > 1, one of the eigenvalues is positive and thus a small deviation from the disease-free equilibrium will grow exponentially until it is no longer small. In this case, the model has an endemic equilibrium at (S*, I*) = (*N*/*R*_0_, (*μ*/*β*)[*R*_0_(1 − *ν*) − 1]), which is stable. The eigenvalues of the Jacobian at this point are

$$\begin{array}{c} \begin{array}{c} \frac{-\mu R_{0}\left(1-\nu \right)}{2}\pm [\frac{{\left(-\mu R_{0}\left(1-\nu \right)\right)}^{2}}{4}-\mu \left(R_{0}\left(1-\nu \right)-1\right)\left(\mu +\gamma \right){]}^{1/2}. \end{array} \end{array}$$ (5)

Putting it all together, when *R*_0_(1 − *ν*) = 1, one of the eigenvalues is zero and there is neither exponential growth nor decay toward the equilibrium point. As *R*_0_(1 − *ν*) decreases below 1 and the distance from the immunization threshold increases, one of the eigenvalues of the disease-free equilibrium becomes more negative and the rate at which perturbations in the associated direction decay increases. Likewise, as *R*_0_(1 − *ν*) increases above 1, at least one of the eigenvalues of the endemic equilibrium becomes greater in magnitude, and the rate at which perturbations in the affected direction move back toward the equilibrium also increases. When the eigenvalues form a complex conjugate pair, the number of infections repeatedly overshoots the equilibrium (*i.e*., the system is underdamped) and the perturbations begin to approach the equilibrium via damped oscillations. In this case, the complex modulus of the eigenvalues measures their magnitude, and it is equal to [*μ*(*μ* + *γ*)(*R*_0_(1 − *ν*) − 1)]^1/2^ = det(**J**)^1/2^. The modulus summarizes both the rate of contraction and the frequency of oscillation of the inward spiral to the equilibrium. In summary, equations for the eigenvalues show that the speed at which perturbations return to the equilibrium increases with |*R*_0_(1 − *ν*) − 1|, the distance of the control parameter to its critical value.

## Observable statistics

**Fig 3:**
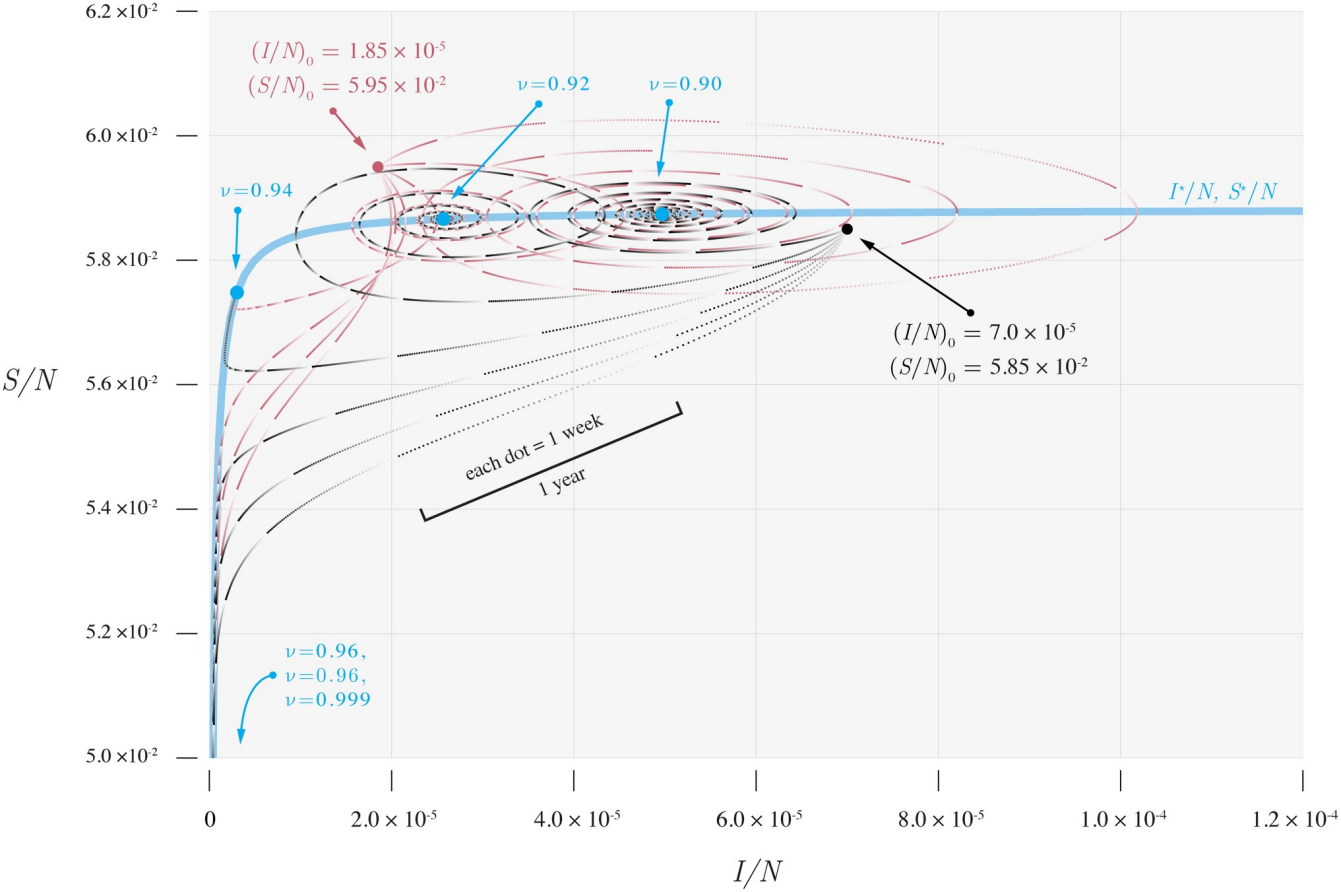
Dynamics of deterministic component of SIR model. Dynamics of the deterministic component of the SIR model (Eq 1) as a function of vaccine uptake. The motion becomes slower as the vaccine uptake approaches the threshold. Parameters are as in Fig 2.

If the trajectories that result from perturbations can be directly observed, as might be the case for data from a large population, these relationships can be used directly to determine the distance to an immunization threshold [8]. Fig 3 illustrates how the motion of perturbations slows as the immunization level approaches the critical level of about 94% (in our example). Consequently, as the immunization level drops to 90%, the total length of the plotted series increases although all the series represent trajectories of perturbations over a period of equal duration. Particularly conspicuous is the increased number of cycles in the trajectories. That is the feature of the dynamics studied in the work of Anderson and May [14, 17]. Although oscillations in both our and their model decline in amplitude over time, a great deal of research has been devoted to explaining why that decay may not occur for real systems. For example, stochasticity and seasonal variation in the transmission rate both have been shown to counteract the damping of oscillations [21–23]. The model-predicted periods of oscillation, then, are also calculations of the imaginary part of the eigenvalue of the Jacobian. To see that, note that when *μ* ≪ *γ* and *R*_0_(1 − *ν*) ≫ 1, the imaginary part of the eigenvalue for our SIR model may be approximated as [*μ*(*R*_0_(1 − *ν*) − 1)*γ*]^1/2^. Also, for small *μ*, the mean age of infection *A* ≈ [*μ*(*R*_0_(1 − *ν*) − 1)]^−1^ [14]. Letting *D* = *γ*^−1^, the expected value of the infectious period, we then have (*AD*)^−1/2^ for the imaginary part of the eigenvalue. Thus we have recovered the expression for the period of 2*π*(*AD*)^1/2^. As the vaccination rate increases and approaches the threshold, the period 2*π*(*AD*)^1/2^ increases.

Likewise, the real part of the eigenvalues for our SIR model at the endemic equilibrium can be approximated as −*μR*_0_(1 − *ν*)/2. As the distance to the immunization threshold |*R*_0_(1 − *ν*) − 1| decreases, the real part of the eigenvalues becomes closer to 0. Because the recovery rate of the amplitude of the oscillations is monotonically related to the real part of the eigenvalues, the recovery of the system from perturbations to its equilibrium slows down. To examine the slowing down, we define a recovery rate as λ = d ln *r*/d*t*, where *r* is the amplitude of the oscillation of (*I* − I*)/*N*. The recovery rate of the amplitude λ decreases as the vaccination rate increases.

Thus, it is now clear how the change of eigenvalues corresponds to the more general slowing of the dynamics as the distance to the immunization threshold |*R*_0_(1 − *ν*) − 1| decreases. This dynamical slowing down is related to the change of both real and imaginary parts of the eigenvalues. The change in the imaginary part results in the lengthening of the period, while the change in the real part affects the recovery rate of the amplitude of the oscillations. Thus, in one sense, the validity of the general approach that relates the speed of a system’s dynamics to its distance to an immunization threshold is well established.

In addition to the distance-speed relationship across populations, there is also some work validating the distance-speed relationship within populations. For example, other work by Anderson and May [24] notes that the period of measles in England and Wales appears to have lengthened to about 3 years following the introduction of extensive immunization in 1968. Curiously, however, the increases in periodicity and average age of infection are not as great as a simple SEIR model would predict [25], perhaps due to strong assortative age mixing [26, 27]. Similarly, whooping cough cycles increased in length in many populations after vaccination [25, 28]. Although this list of examples is short, it nevertheless shows the phenomenon of critical slowing down to be sufficiently robust to appear in data from natural populations.

## Anticipating emergence and re-emergence

The aim of the present article is to suggest ways by which these behaviors in near-critical systems illuminate the approach to epidemic transitions [20, 29]. Showing that observable summary statistics behave in characteristic ways in parametric models provides grounds for measuring the same statistics in data. One way to find more examples of distance-speed relationships could be to use a model that explicitly accounts for the effects of random noise on system dynamics [30]. For small populations, particularly, one expects the intrinsic noise from demographic stochasticity to play an important role [16, 21, 30]. The decay of individual perturbations in such a system are obscured from direct observation by the noise, but their slowing down can be deduced from distributional properties of the time series [20, 29, 31].

**Fig 4:**
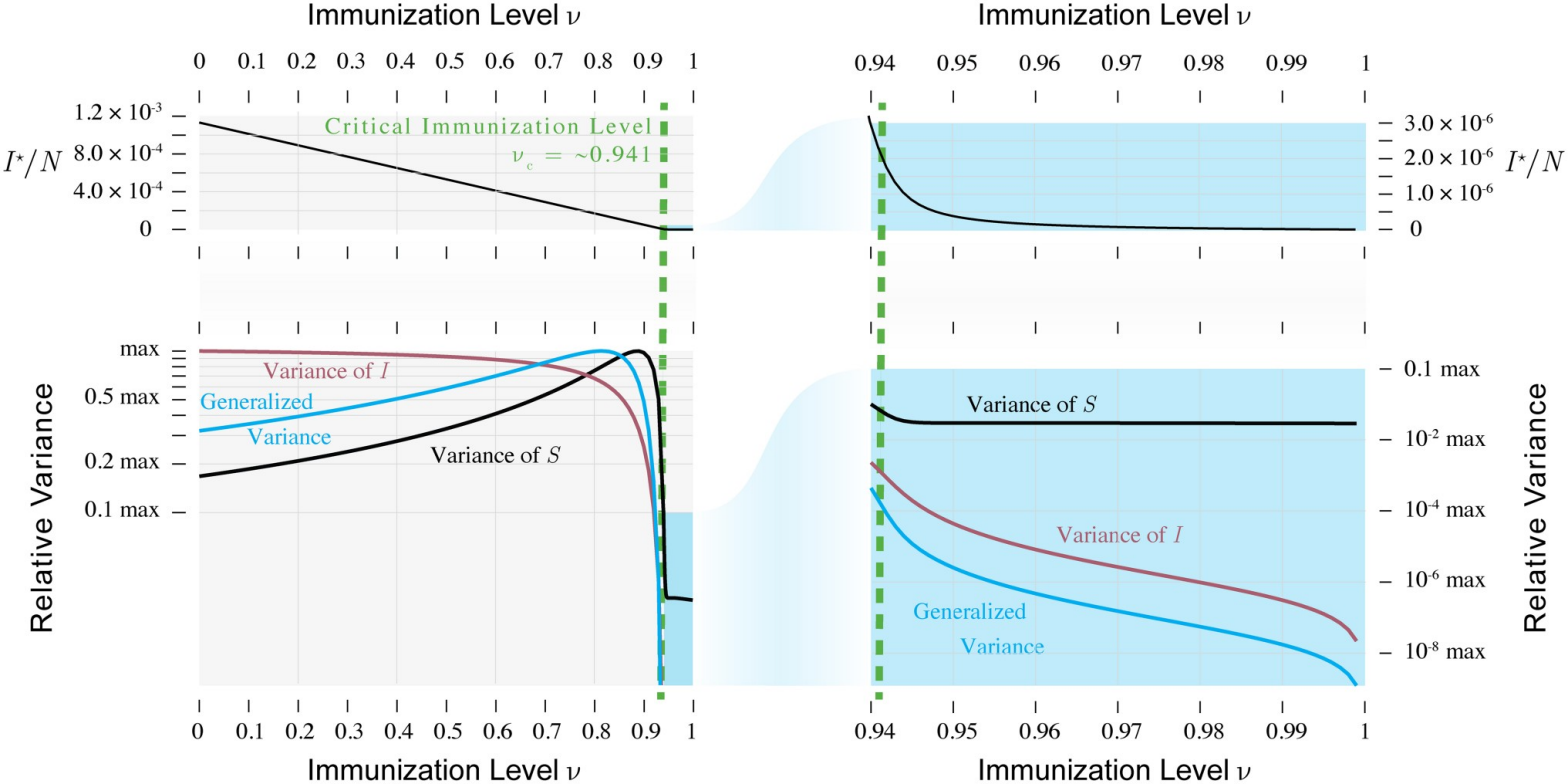
Variance as a function of vaccine uptake. The variance of *S* and the generalized variance peak near the threshold, whereas the variance of *I* always decreases as vaccine uptake increases. The right panel shows that the variance of *S* would not be as informative as that of *I* for an approach to the threshold from above. Parameters are as in Fig 2.

**Fig 5:**
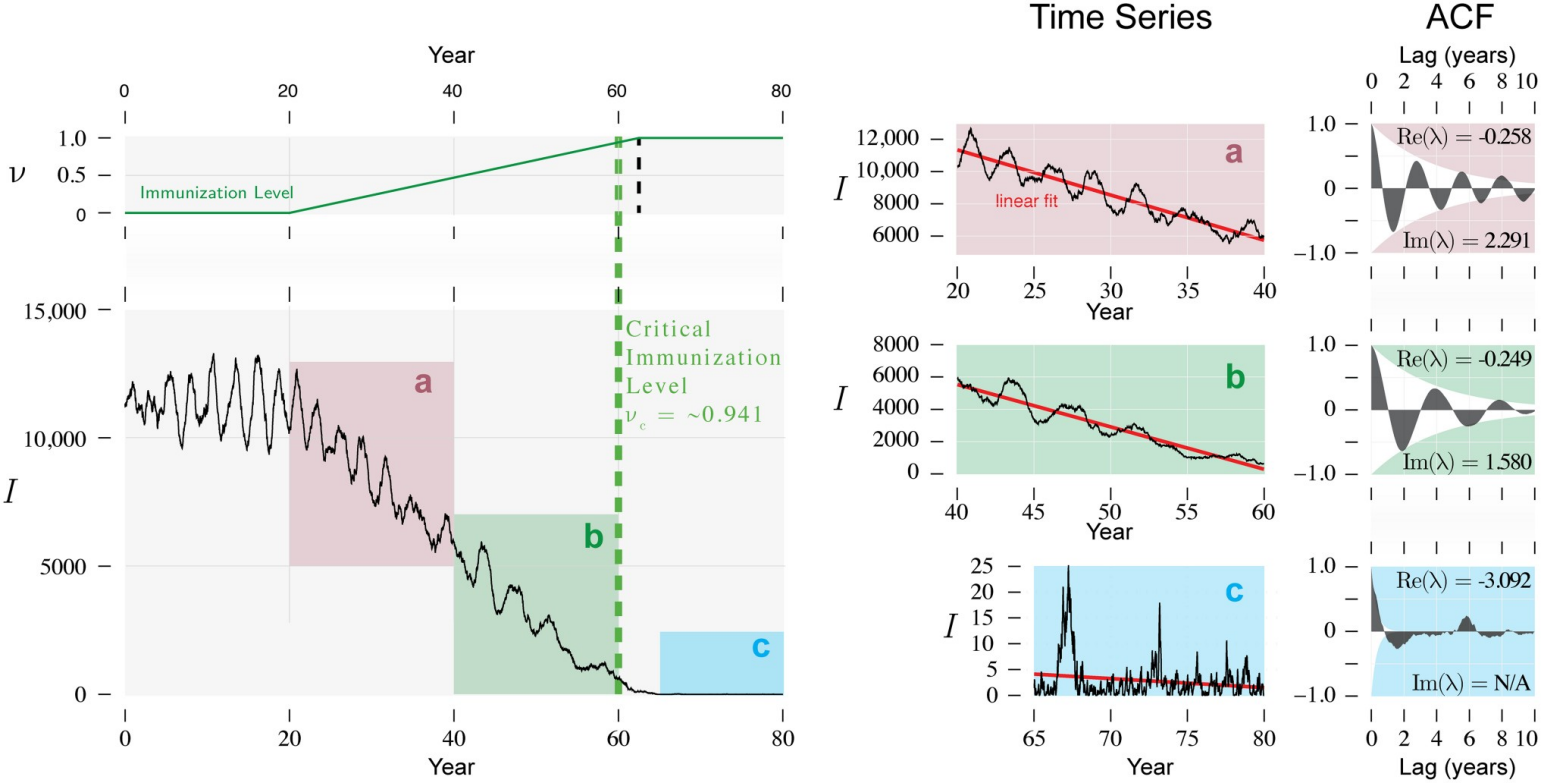
Dynamics of the number infected in a slow approach to elimination. Critical slowing down does not lead to an increase in the variance of this variable as the immunization threshold is approached. However, critical slowing down can still be observed from the decrease in the frequency of oscillations in the autocorrelation function (ACF). Vaccine uptake *ν* increasing 0.025/year from *ν* = 0 in year 20. Other parameters are as in Fig 2.

One potentially useful distributional property is the variance, which arises from the balance between the random noise that generates perturbations (the Brownian motion terms in the model) and the rate at which perturbations decay toward the equilibrium [32]. We have already seen that, in at least one direction, the rate of decay approaches zero as a threshold is approached, caused by either the dominant eigenvalue, or the real part of the dominant eigenvalue, approaching 0. Unless the noise decreases equally fast, the system is unable to as efficiently dissipate perturbations and variation accumulates. This implies that for demographic stochasticity in a population not undergoing major changes in size, any changes in the variance of the noise are typically much smaller than the changes in this relaxation. Consequently, the variance of the time series tends to peak when the control parameter is near the threshold of a model [20, 29, 33]. However, for a multivariate model such as our SIR model, the variance of the time series for individual variables can behave much differently [34]. Figs 2, 4 and 5 show that the variance of the number infected always decreases with the vaccine uptake, whether or not the threshold is being approached. This is because the variance of the perturbations to *I* increases with vaccine uptake enough to wash out the signal of changes in stability. On the other hand, the variance of *S* peaks closer to the critical immunization level. One implication of this property is that changes in the variance of *S* could be more useful for monitoring an approach to the critical level.

**Fig 6:**
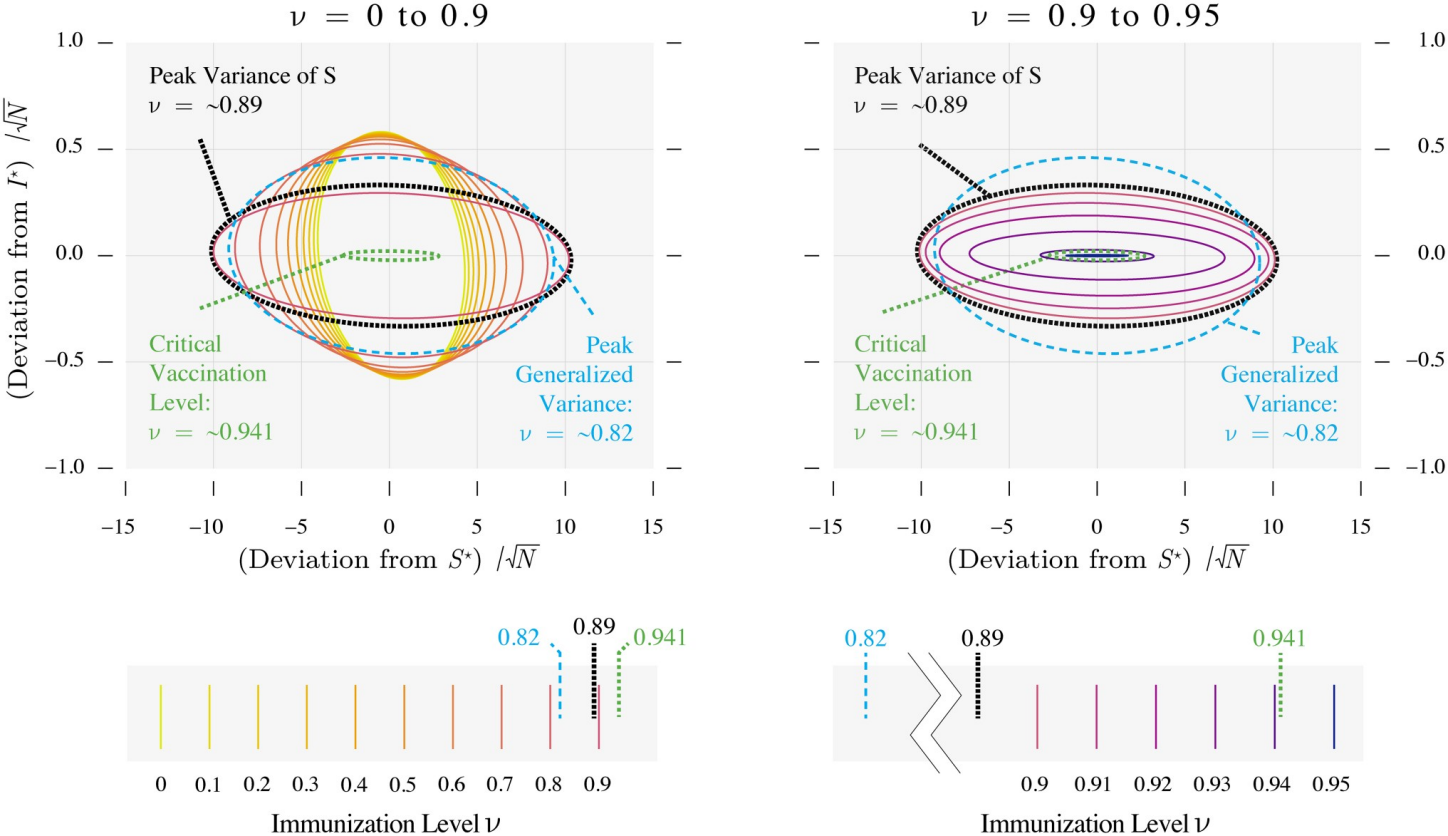
Bivariate spread of the deviations from the equilibrium as a function of vaccine uptake. The ellipses indicate the area containing the deviations 95% of the time. The area of the ellipse is largest in the vicinity of the threshold immunization level, which is consistent with the common result that critical slowing down leads to increases in variance. Parameters are as in Fig 2.

One might also expect that in a multivariate model a multivariate summary of variance would peak near the threshold, based on the fact that the variance should be very high along the eigendirection associated with the eigenvalue that approaches zero, which should dominate a multivariate summary of the variance. Because the stationary distribution of the linearized model is bivariate normal [13], we can visualize the bivariate spread of the data as an ellipse that represents the smallest region that contains 95% of observed deviations of *S* and *I* from their equilibrium values. Fig 6 plots these regions for a range of immunization levels. One can see that the regions are largest between the points when the variance in *S* is maximized and when the variance of *I* is maximized. One numerical summary of these features is the generalized variance [35], defined as the determinant of the variance-covariance matrix, which is proportional to the area of the ellipses squared. Fig 4 shows that the generalized variance also peaks somewhat close to the threshold, although not as close as does the variance of *S*. We have noticed that these peaks come closer to the threshold as the *per capita* sparking rate *η* is reduced, but even in the limit that *η* = 0 their peaks lie at immunization levels slightly below the threshold. Thus when approaching the immunization threshold from below, trends in the generalized variance or the variance of *S* are most likely to increase when the threshold is relatively distant. They are then perhaps best suited to monitoring the progress of an immunization program in its early stages. The variance could also be useful as an early warning signal for disease re-emergence resulting from declining vaccine uptake. Fig 4 shows that the variance of *S*, *I*, and also the generalized variance accelerates upwards as the immunization threshold is approached from above. In summary, for vaccine-preventable diseases that are aptly modeled by an SIR model near equilibrium subject to regular perturbations by intrinsic noise, trends in the variance of a time series can be useful indicators of trends in vaccine uptake.

A second potentially useful distributional property is the autocorrelation. The autocovariance of small deviations from the equilibrium at a given lag is given by the product of the covariance matrix and the matrix exponential of the Jacobian times the lag [13]. It follows that the autocorrelation function has the form of a mixture of exponential functions of the lag multiplied by the eigenvalues. In general, the mixture of the exponentials is different for *S* and *I* and it depends on how the eigendirections of the Jacobian project onto each variable and also on the covariance matrix of the noise that generates perturbations of the system. Importantly, for a model excited by intrinsic noise and controlled by vaccination, the decay rate of the autocorrelation of *I*, and not that of *S*, is often close to the value of the eigenvalue that crosses zero at the immunization threshold. Thus the autocorrelation of *I* at a given lag often increases when vaccine uptake is declining toward the threshold value.

Another case of interest is when the disease is endemic but vaccine uptake is rising. In this case, the system is often underdamped, and the autocorrelation exhibits damped oscillations as the lag increases. The imaginary part of the eigenvalues can then be estimated by the frequency of the oscillation and the real part of the eigenvalue by the damping rate. Observations of either the *S* or *I* variable can be used to estimate the autocorrelation function. Fig 5 illustrates how an autocorrelation function of this form changes with increasing vaccine uptake. It is also clear from the figure that the time series of *I* itself oscillates with a characteristic frequency. However, in the presence of intrinsic noise the dominant frequency of the time series of *I* does not in general have a simple relationship with the eigenvalues of the Jacobian [16]. This implies that estimating both the damping and the periodicity of the autocorrelation function may provide a more reliable estimate of the distance to the immunization threshold than simply identifying the dominant frequency of the time series of the number infected.

The variance and autocorrelation are but two of a growing number of distributional properties that are expected to change in predictable ways as a threshold is approached [29, 36, 37]. Thresholds—not just in the SIR model that we have used as an example—for many types of bifurcations in models of different systems have an underlying deterministic component. Indeed, the apparent versatility of these methods combined with the difficulty of pinning complex systems down to a specific model has made them the subject of much current research. It is reasonable to expect that the approach to epidemic transitions, including catastrophic transitions, in a wide variety of transmission systems may be estimable from the observable dynamics of the system, in a manner similar to what we have described using a stochastic SIR model. It also seems reasonable that, as we have seen for the SIR model, the signals of the approaching bifurcation may sometimes be complicated and depend on the observation of certain key variables [34]. The benefit of working out such details for more realistic models could be improvements in our ability to predict epidemics of emerging infectious diseases.

## Conclusions

Many factors can result in the emergence and re-emergence of infectious diseases, including collective changes in individual movement patterns, population-level birth rates, pathogen evolution and, most poignantly, declines in vaccine uptake [38]. Consequently, re-emergence occurs regularly in populations around the world [39]. Although infectious disease forecasting is an active area of research [40–43], methods for anticipating disease emergence and re-emergence remain under-developed, hampering preparedness, surveillance and detection, and the development of response strategies. Further, although the study of infectious disease dynamics is very mature and a great many diagnostic, informatic, and simulational tools exist for understanding the course of epidemics, these tools are of little use prior to the onset of sustained transmission [44]. The problem is further complicated by the wide variety of conditions under which emerging pathogens arise and the knowledge gap that exists prior to emergence concerning routes of infection, rates of transmission, mechanisms for persistence, roles of social and commercial networks, and functions of transportation systems and cultural practices in the dissemination of novel pathogens [5, 45–47].

For systems in which transmission is relenting (*R*_0_ tending to 1 from above), documenting paths to disease elimination is valuable and has been identified as a critical component of elimination of several tropical diseases [48]. However, as with emergence scenarios, predicting elimination has proven more formidable than anticipated, due to the different transmission processes that dominate the final decline to extinction [49, 50]. A model-independent method for anticipating disease emergence and elimination that may be applied to a wide range of scenarios would therefore be of considerable value. Based on the theory of critical slowing down, we are now developing a family of online algorithms for early warning of infectious disease emergence/re-emergence and leading indicators of elimination that take advantage of dynamical properties that infectious disease systems exhibit as they approach and cross a tipping point [29, 51]. In contrast to detection systems [52, 53], our theory (and associated toolkit) aims to forecast epidemic transitions before they occur. Complementing the highly detailed network and agent-based models developed by others [54, 55], these approaches exploit universal, qualitative properties of contagion systems [20]. While these methods are most informative when the transmission kinetics are sufficiently well understood, this is, reliable mechanistic models may be fit [44, 56], the model-independent approach also works under varying conditions of ignorance and uncertainty [31, 57].
